# Study of the reusability and stability of nylon nanofibres as an antibody immobilisation surface

**DOI:** 10.3762/bjnano.15.8

**Published:** 2024-01-15

**Authors:** Inés Peraile, Matilde Gil-García, Laura González-López, Nushin A Dabbagh-Escalante, Juan C Cabria-Ramos, Paloma Lorenzo-Lozano

**Affiliations:** 1 Biological Defence Area, Department of NBC Defence Systems and Energetic Materials, National Institute for Aerospace Technology “Esteban Terradas” (INTA)-Campus La Marañosa, Ctra. M-301, Km 10, 28330, San Martín de la Vega, Madrid, Spain

**Keywords:** biosensor, biothreat, immunodetection, nanofibre, nylon

## Abstract

In the case of a biological threat, early, rapid, and specific detection is critical. In addition, ease of handling, use in the field, and low-cost production are important considerations. Immunological devices are able to respond to these needs. In the design of these immunological devices, surface antibody immobilisation is crucial. Nylon nanofibres have been described as a very good option because they allow for an increase in the surface-to-volume ratio, leading to an increase in immunocapture efficiency. In this paper, we want to deepen the study of other key points, such as the reuse and stability of these nanofibres, in order to assess their profitability. On the one hand, the reusability of nanofibres has been studied using different stripping treatments at different pH values on the nylon nanofibres with well-oriented antibodies anchored by protein A/G. Our study shows that stripping with glycine buffer pH 2.5 allows the nanofibres to be reused as long as protein A/G has been previously anchored, leaving both nanofibre and protein A/G unchanged. On the other hand, we investigated the stability of the nylon nanofibres. To achieve this, we analysed any loss of immunocapture ability of well-oriented antibodies anchored both to the nylon nanofibres and to a specialised surface with high protein binding capacity. The nanofibre immunocapture system maintained an unchanged immunocapture ability for a longer time than the specialised planar surface. In conclusion, nylon nanofibres seem to be a very good choice as an antibody immobilisation surface, offering not only higher immunocapture efficiency, but also more cost efficiency as they are reusable and stable.

## Introduction

Biological threats involve a wide range of risks not only to the human population, but also to livestock and crops [1], affecting both human health (mortality, morbidity, and incapacity) and the economy (crop failures, livestock deaths, and investments in health and safety) [2]. For this reason, early, rapid, and specific detection of biological threats becomes a very important objective to react as early as possible. Many efforts have been made in this direction. When designing a new sensor device, not only the rapid and specific identification has to be taken into account, but also ease of handling, on-site use, and low production cost. Thus, several authors, such as Janik-Karpinska and colleagues in 2022 [3], have pointed out that rapid detection of pathogens and toxins in food, water, and the environment is a paramount health and safety need to reduce the risk of pandemic contamination. Early, reliable, and accurate diagnosis is therefore essential for health and food safety [4–5].

In this context, immunodetection seems to be a very good option [6]. There are many applications of immunoassay devices in health, food industry, and clinical applications. Immunoassay devices have been used not only for the detection of bacteria and viruses [7], but also for the measurement of drugs [8] and hormones [9], or for the determination of glucose in urine [10].

The specificity of antigen–antibody binding and how the antibody is attached to the biosensor surface, in terms of density, orientation, and stability, will determine the diagnosis capability of the device [11]. Thus, the immobilisation surface of the device is one of the key points in the development of new sensors.

Nylon has been used as immobilisation surface in numerous applications, such as the immobilisation of enzymes and microorganisms [12–13], and the immobilisation of antibody in enzyme immunoassays [14]. Nylon 6 (or polyamide 6, PA6) nanofibers (NFs) have been used as an immobilisation surface in biosensors [15]. Efficiency studies of nanofibres manufactured by electrospinning have been carried out in our laboratory, determining the optimal nanofibre thickness regarding stability and biofunctionalisation [16]. Our results showed that the NFs’ surface provides advantages over a planar nylon surface in terms of increased immunocapture efficiency as the higher surface area/volume ratio in the nanofibre allows for a greater amount of immobilised antibody in the same space [17]. In addition, some studies demonstrate the suitability of electrospun nylon NFs for the development of Fabry–Pérot-based optical biosensors [18–19]. However, for the selection of such NFs as immobilisation surfaces in biosensors, it seems necessary to study those characteristics of the immobilisation surface that contribute to their lower cost.

In this regard, this paper not only investigates the reuse of NFs, but also whether this immobilisation surface provides a longer life for an immunocapture system. These characteristics are key points to obtain a more cost-effective and environmentally friendly immobilisation surface.

One of the aims of developing a rapid and easy-to-use biosensor is to be able to detect a biological threat as early as possible. In the “Nanofibre reusability study” developed in this paper, bovine serum albumin (BSA) was chosen as a surrogate for biotoxins. In contrast, in the “Stability study” carried out in this paper, ricin was used as a representative biotoxin instead of a surrogate because the “Stability study” required less handling than the “Nanofibre reusability study”. Ricin has been chosen as a representative biotoxin because it has been used in biological warfare attacks because of its high toxicity, stability, and availability. It belongs to the ribosome-inactivating protein family and causes cell death by disrupting protein synthesis [20].

## Results and Discussion

### Results of nanofibre reusability study

#### High-salinity antigen/antibody (Ag/Ac) elution buffer pH 6.6 as stripping agent

**Figure 1:**
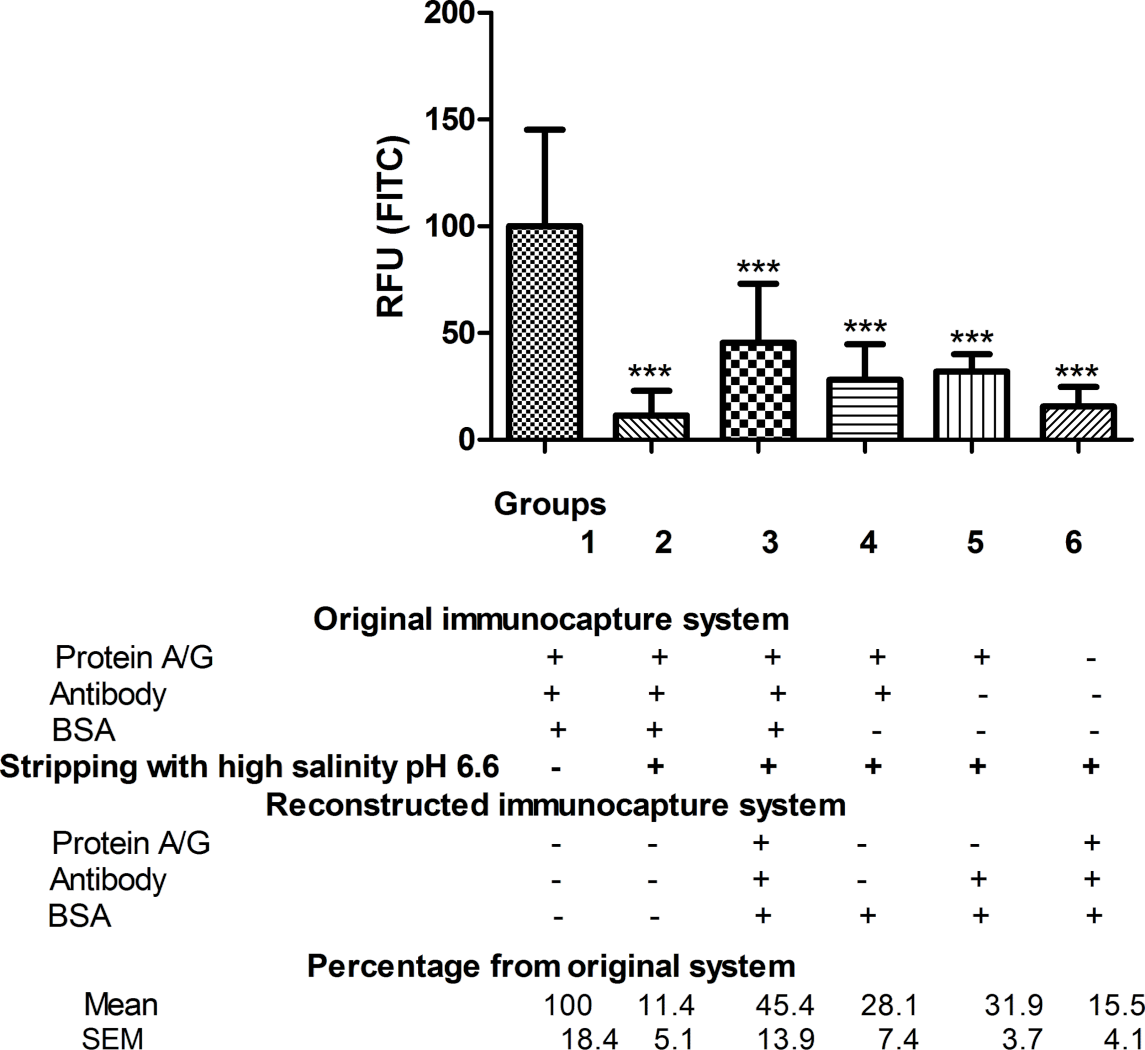
FITC fluorescence of anti-BSA antibody. For each group, the FITC fluorescence data of the immobilised anti-BSA antibody, measured in RFU, are given as percentages relative to the reference group 1, *n* = 5–6. Stripping treatment with commercial Ag/Ac elution buffer pH 6.6 was performed in all groups except group 1, which was used as the reference in the statistical analysis. One-way ANOVA followed by Newman–Keuls test. Difference from original immunocapture system fluorescence (reference group: group 1): ***p < 0.001.

A commercial Ag/Ac elution buffer pH 6.6 with high salinity was able to remove almost all antibody fixed on the nanofibres through protein A/G (88.6%). The retained antibody fraction after stripping treatment (group 2) was only 11.4% compared to the reference group 1, which is 100% (Figure 1).

The amount of bound antibody is indicated by the amount of fluorescein (FITC) fluorescence detected as this fluorochrome is associated with the antibody in question. It is measured as relative fluorescence unit (RFU) (index explained in the Experimental section). Results are expressed as the mean RFU of the replicates, and the variation is expressed as the standard error of the mean (SEM).

When the immunocapture system had been reconstituted after the stripping procedure (group 3), only 45.4% of bound antibody was found, compared to the total amount of antibody fixed in group 1 (Figure 1). This suggests that antibody binding was altered by the buffer. The same results were found when BSA alone (group 4) or antibody plus BSA (group 5) was administered after stripping treatment, yielding 28.1% and 31.9%, respectively, of the amount of antibody fixed in group 1 (Figure 1).

Bare NFs (group 6) are NFs that have undergone the stripping process without prior binding to the immunocapture system. These bare NFs were damaged to the extent that they were unable to bind the immunocapture system (15.5% compared to the total antibody fixed in group 1 (Figure 1). Hence, it seems that commercial Ag/Ac elution buffer pH 6.6 with high salt content damages the nylon nanofibers, thereby altering their immunocapture ability.

Having studied how the amount of immobilised antibody was affected, we also wanted to determine how the immunocapture capacity of these immobilised antibodies was affected. This was determined by assessing the fluorescence associated with the immunocaptured antigen, which, in this study, was the protein toxin simulant BSA. The BSA-associated fluorochrome was phycoerythrin (RPE), and the amount of immunocaptured antigen was assessed by the intensity of RPE fluorescence (measured in RFU).

**Figure 2:**
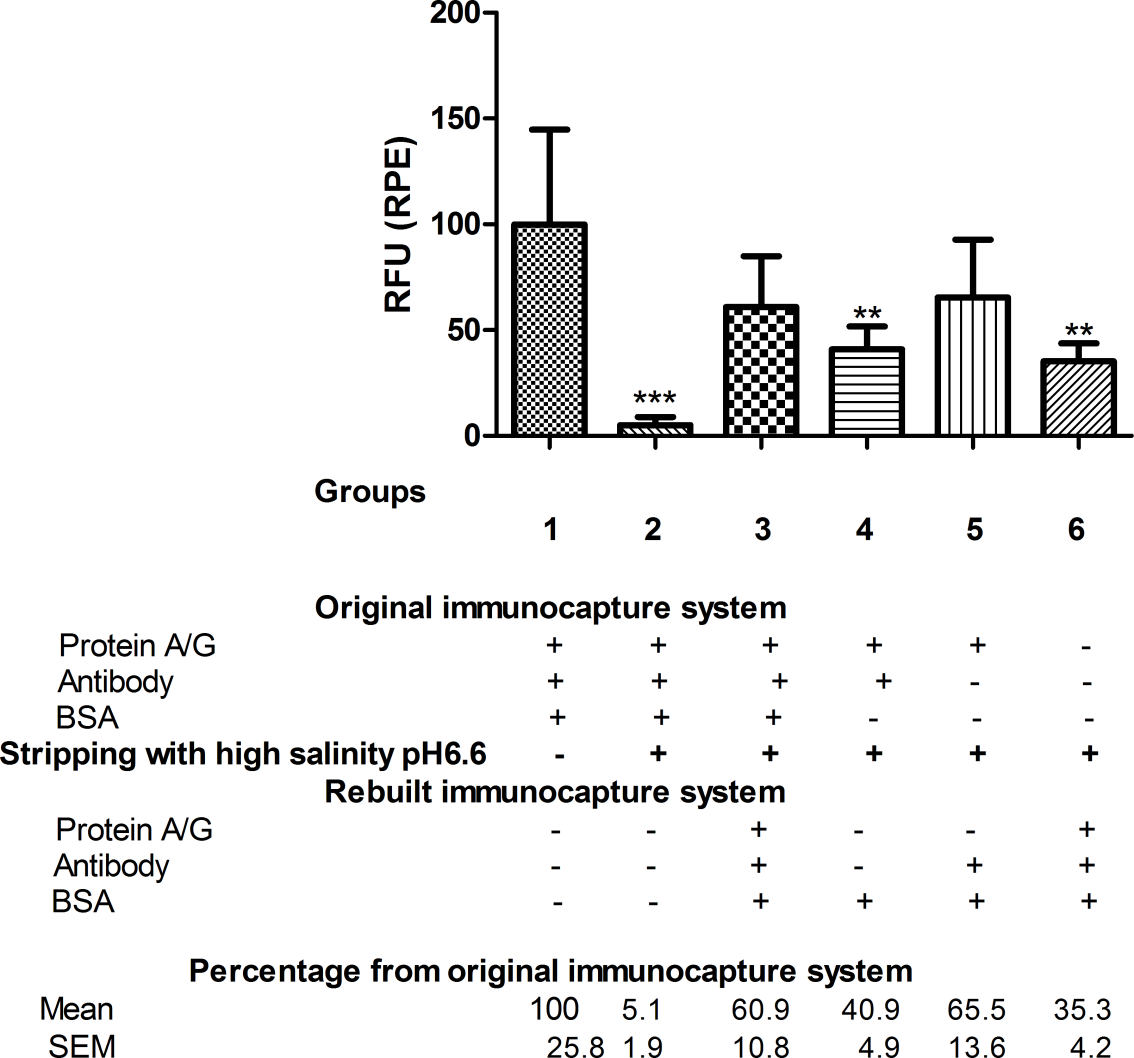
RPE fluorescence of immunocaptured BSA. For each group, the RPE fluorescence data of the immunocaptured BSA, measured in RFU, are given as percentages relative to the reference group 1, *n* = 5–6. Stripping treatment with commercial Ag/Ac elution buffer pH 6.6 was performed in all groups except group 1, which was used as the reference in the statistical analysis. One-way ANOVA followed by Newman–Keuls test. Difference from original immunocapture system fluorescence (group 1): ***p < 0.001, **p < 0.01.

Regarding the BSA immunocapture, the results showed some unspecific BSA binding after stripping treatment (group 3) as the amount bound antibody (45.4%) was less than that of immunocaptured BSA (60.9%), both values compared to group 1 (Figure 1 and Figure 2).

Similar results were obtained in the reconstituted immunocapture system when BSA alone (group 4) or antibody plus BSA (group 5) were administered after stripping. While 28.1% and 31.9% of bound antibody was found in groups 4 and 5, respectively, 40.9% and 65.5% of immunocaptured BSA was detected in these groups, both compared to group 1 (Figure 1 and Figure 2). Furthermore, after stripping treatment, the bare nanofibre (group 6) was only able to bind 15.5% of the total antibody initially bound, whereas 35.3% of the BSA was immunocaptured in this group (group 6) compared to group 1 (Figure 1 and Figure 2).

#### Ammonium hydroxide buffer pH 11 as stripping agent

**Figure 3:**
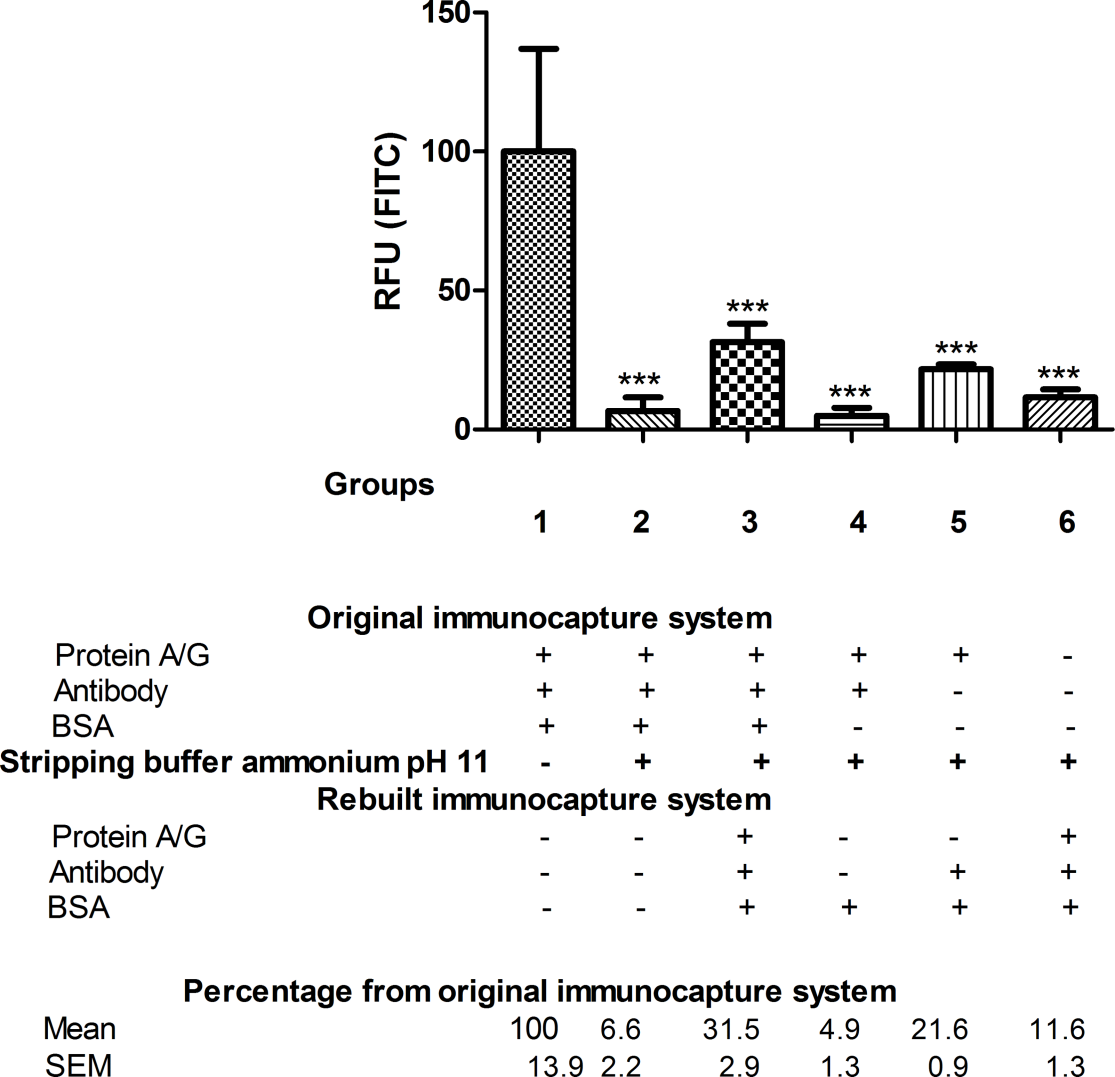
FITC fluorescence of anti-BSA antibody. For each group, the FITC fluorescence data of the immobilised anti-BSA antibody, measured in RFU, are given as percentages relative to the reference group 1, *n* = 5–6. Stripping treatment with ammonium hydroxide buffer pH 11 was performed in all groups except group 1, which was used as the reference in the statistical analysis. One-way ANOVA followed by Newman–Keuls test. Difference from original immunocapture system fluorescence (group 1, reference group): ***p < 0.001.

Ammonium hydroxide buffer pH 11 gave similar results to the commercial high-salinity Ag/Ac elution buffer pH 6.6 (Figure 3).

This treatment was able to remove almost all of the antibody captured by the NFs via protein A/G (93.4%) since the retained antibody after stripping treatment (group 2) was only 6.6% compared to group 1 (Figure 3). However, the ammonium hydroxide buffer pH 11 interfered with the reconstituted immunocapture system to such an extent that only 31.5% of the captured antibody was detected after the reconstruction process (group 3) compared to group 1 (100%). When bare NFs were treated with the ammonium buffer (group 6), almost no antibody was bound (11.6%) (Figure 3).

**Figure 4:**
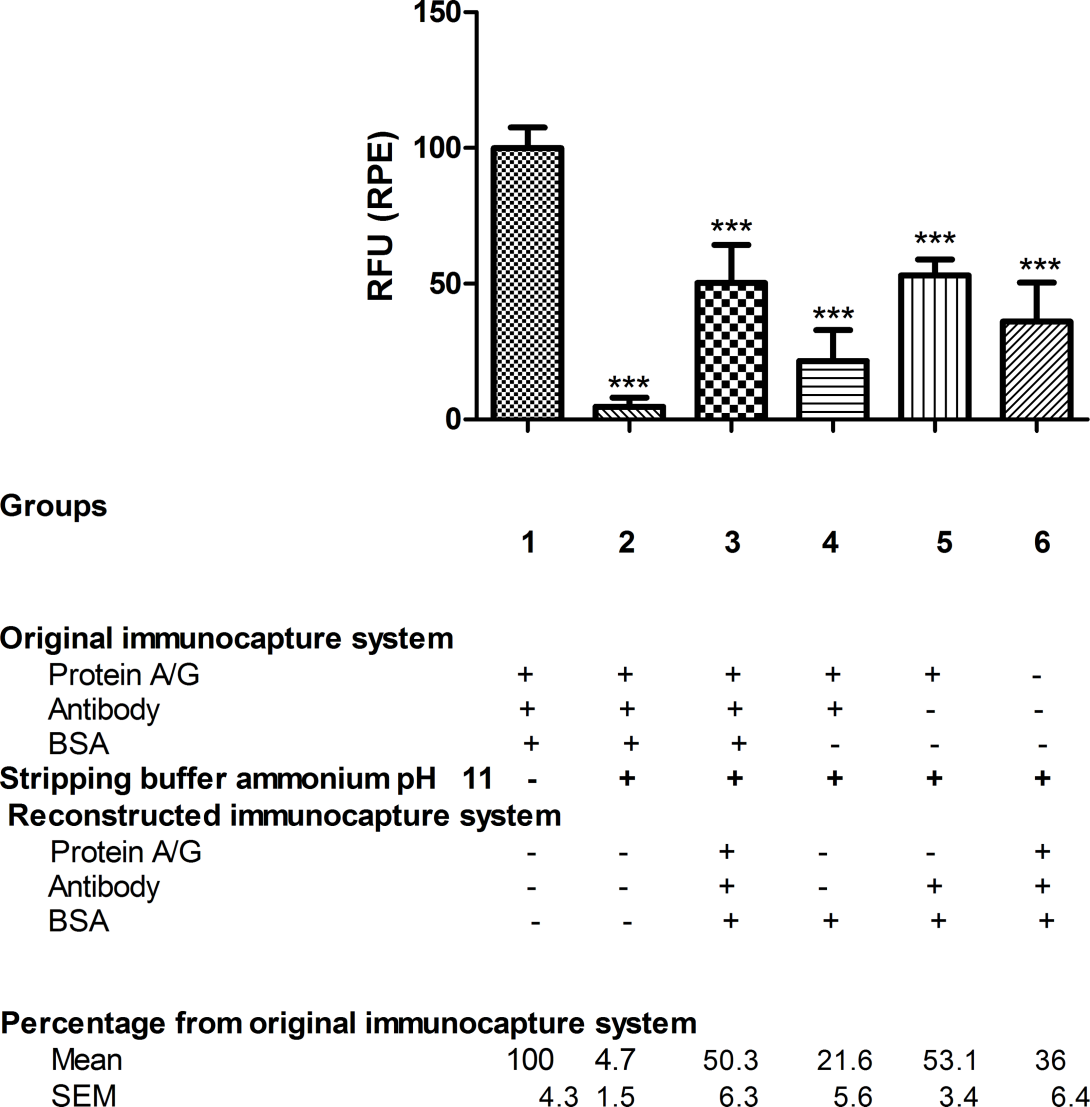
RPE fluorescence of immunocaptured BSA. For each group, the RPE fluorescence data of the immunocaptured BSA, measured in RFU, are given as percentages relative to the reference group 1, *n* = 5–6. Stripping treatment with ammonium hydroxide buffer pH 11 was performed in all groups except group 1, which was used as the reference in the statistical analysis. One-way ANOVA followed by Newman–Keuls test. Difference from original immunocapture system fluorescence (group 1, reference group): ***p < 0.001.

As with the commercial high-salinity Ag/Ac elution buffer pH 6.6, non-specific binding of BSA was observed in NFs after stripping treatment (group 3) as more immunocaptured BSA was detected than bound antibody (Figure 4).

In group 3, while 31.5% of bound antibody was detected, 50.3% of BSA was immunocaptured, both values compared to group 1 (Figure 3 and Figure 4). After stripping, the reconstituted immunocapture systems after reapplying BSA only (group 4) or antibody plus BSA (group 5) exhibited 4.9% and 21.6% of bound antibody and 21.6% and 53.1% of immunocaptured BSA, respectively, compared to group 1 (Figure 3 and Figure 4). Furthermore, after buffer treatment, the bare NFs (group 6) were able to bind only 11.6% of the total antibody and immunocaptured 36% of BSA (Figure 3 and Figure 4).

Thus, both ammonium hydroxide and commercial elution buffer had a detrimental effect on the nylon NFs. Hence, neither of these well-known solutions should be used as stripping buffers with these NFs.

#### Glycine buffer pH 2.5 as stripping agent

**Figure 5:**
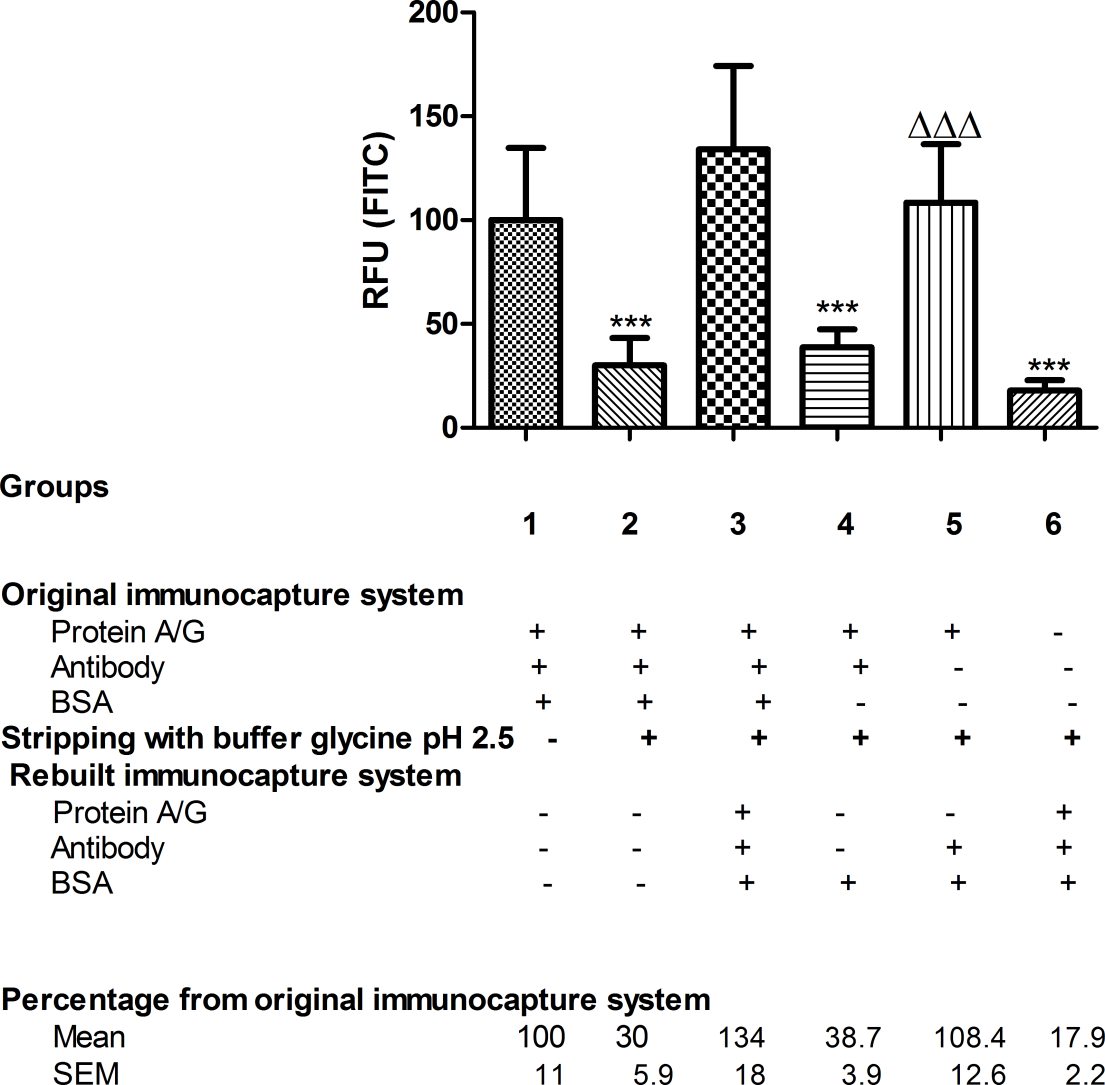
FITC fluorescence of anti-BSA antibody. For each group, the FITC fluorescence data of the immobilised anti-BSA antibody, measured in RFU, are given as percentages relative to the reference group 1, *n* = 5–6. Stripping treatment with glycine buffer pH 2.5 was performed in all groups except group 1, which was used as the reference in the statistical analysis. One-way ANOVA followed by Newman–Keuls test. Difference from original immunocapture system fluorescence (group 1): ***p < 0.001. Difference from bare nanofibers after stripping and reconstructing the immunocapture system (group 6): ΔΔΔ*p* < 0.001.

In contrast, buffer containing glycine pH 2.5 was able to remove 70% of the total fixed antibody since the retained antibody after stripping treatment (group 2) was 30% compared to the total antibody bound in group 1 (Figure 5).

**Figure 6:**
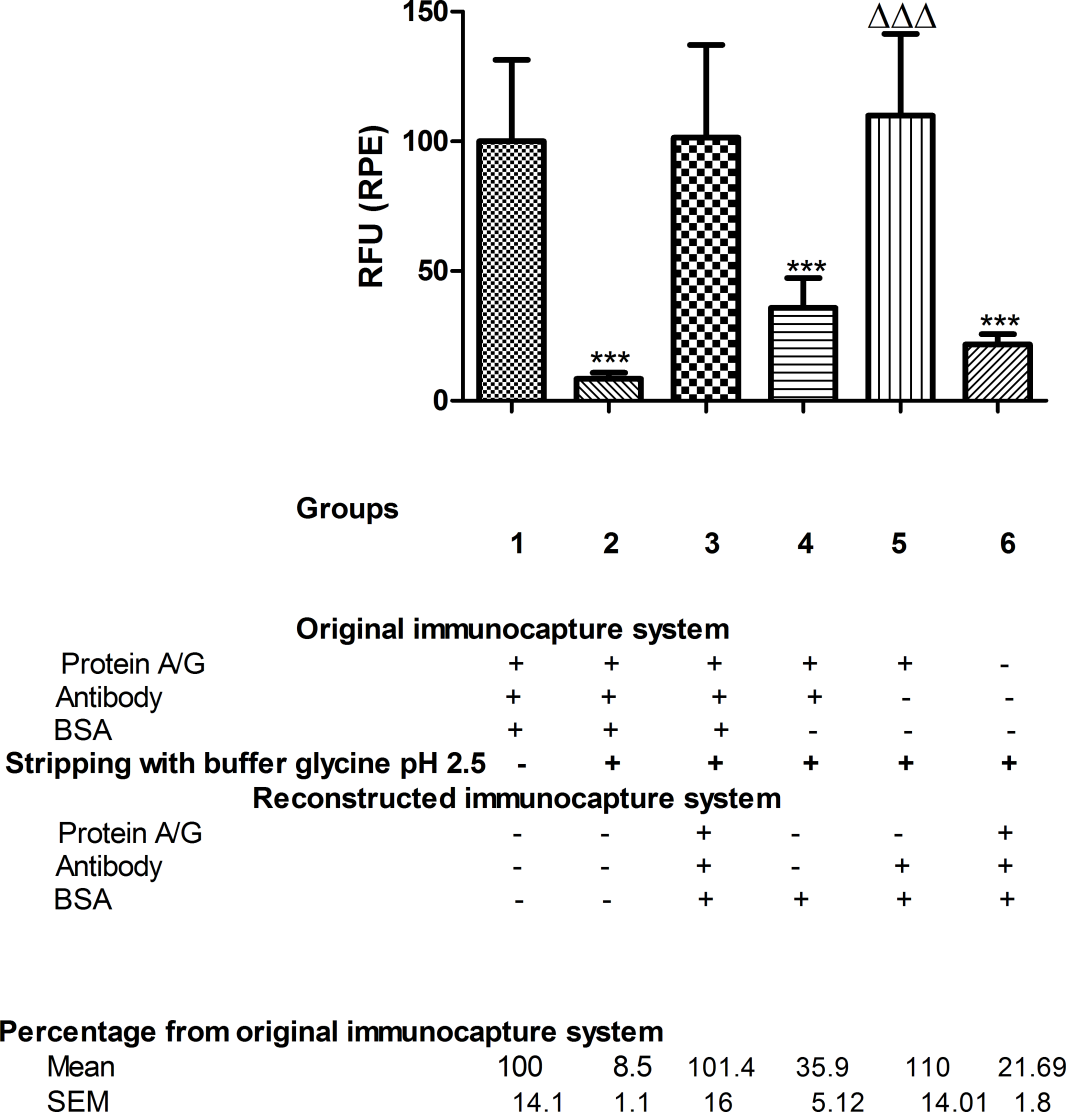
RPE fluorescence of immunocaptured BSA. For each group, the RPE fluorescence data of the immunocaptured BSA, measured in RFU, are given as percentages relative to the reference group 1, *n* = 5–6. Stripping treatment with glycine buffer pH 2.5 was performed in all groups except group 1, which was used as the reference in the statistical analysis. One-way ANOVA followed by Newman–Keuls test. Difference from original immunocapture system fluorescence (group 1, reference group): ***p < 0.001. Difference from bare nanofiber after stripping and reconstructing the immunocapture system (group 6): ΔΔΔ*p* < 0.001.

When the immunocapture system was rebuilt again after stripping (group 3), the amount of bound antibody (134%) was similar to that of total antibody bound before stripping (group 1, 100%) (Figure 5). It was also consistent with the BSA immunocapture results as the reconstituted immunocapture system (group 3) was able to bind the same amount of BSA (101.4%) as the immunocapture system before stripping (group 1, 100%) (Figure 6).

After glycine pH 2.5 treatment, when BSA was administered alone (group 4), only 35.9% of immunocaptured BSA was detected. The same percentage of antibody was detected after the stripping treatment (38.7%), both compared to group 1 (Figure 5 and Figure 6). When antibody was re-administered after stripping (group 5), the antibody was again fully bound (108.4% of bound antibody compared to group 1) (Figure 5), and BSA was immunocaptured in the same way (110%) (Figure 6). This suggests that only antibody, but not protein A/G, was eluted from the NFs.

In contrast to the previous treatments, no unspecific BSA binding was found with NFs treated with glycine pH 2.5 as both antibody and immunocaptured BSA showed the same percentage values compared to group 1.

Another interesting finding was that glycine buffer pH 2.5 damaged the bare NFs by rendering them unable to bind to the immunocapture system (group 6). They were only able to bind 17.9% of the total antibody compared to group 1 (Figure 5). However, when protein A/G was anchored prior to treatment with glycine buffer pH 2.5 (group 5), the rebuilt immunocapture system showed the same rates of antibody immobilisation (108.4%) and immunocaptured BSA (110%) as group 1 (Figure 5 and Figure 6). This suggests that protein A/G protects the NFs from damage by the glycine buffer pH 2.5.

### Discussion of nanofibre reusability

This study has shown that the effect of the pH value on protein A/G is very significant. A strong acid (pH 2.5) caused protein A/G to dissociate from the antibody, but not from the NFs. A higher pH value, such as ammonium buffer pH 11, caused the protein A/G to dissociate and/or not to anchor to the nylon nanofibres. The same results were obtained with the high-salinity commercial Ag/Ac elution buffer pH 6.6, which operates under near-neutral conditions but has a high salt content.

The structures of protein A/G and nylon and their interactions may explain all these results. On the one hand, protein A/G binds to the constant fraction (Fc) of the antibody by hydrophobic interactions through binding sites inside of its three-dimensional structure [21–22]. The polar side chains are located on the outside of the protein molecule, allowing the protein to form hydrogen bonds with nylon. On the other hand, nylon is a polyamide that contains amide groups and free amine groups at the ends of its polymer chains, as well as carboxyl groups. These amide and amine groups provide excellent hydrogen bonding sites [23–24].

Regarding the binding of antibody to protein A/G, it has been described that this occurs at pH values between 5 and 8 because of hydrophobic interactions [21–22]. Acidic pH values below 5 cause protein A/G to separate from antibody, probably by imposing positive charges on amino acids with p*K*_a_ values above 5, such as histidine, as described in Zarrineh et al. for the interaction between protein A and the Fc of antibody [25]. Our results are consistent with this; a strong acidic pH, such as glycine buffer pH 2.5, caused protein A/G to dissociate from antibody.

Protein A/G was dissociated from nylon under basic pH conditions such as ammonium buffer pH 11. As the isoelectric point (pI) of protein A/G is 4.65, there is a higher percentage of acid groups, such as aspartic acid and glutamic acid. These aminoacids have carboxylic acid groups in their side chains, which lose protons at pH values higher than their p*K*_a_ and become negatively charged as a result. In addition, nylon is negatively charged at basic pH [26]. This is understandable as nylon is a polyamide that contains not only many amide groups and free amine groups at the ends of its polymeric chains, but also a large number of carboxyl groups, more than amine groups, which give the nanofibres a negative charge in the basic pH range [23]. Therefore, basic pH levels such as pH 11, but not strongly acidic pH levels such as 2.5, could impart a negative charge to the carboxylic acid groups in both protein A/G and nylon NFs, preventing hydrogen bonds between them.

Acidic pH, such as pH 2.5, does not alter the binding of protein A/G to nylon. However, bare nylon nanofibres were found to be altered by this treatment. This is understandable as polyamides, although containing both negative and positive centres, have amide and amine groups, which are protonated at acidic pH [23]. When protein A/G was administered prior to glycine buffer pH 2.5, no effect was observed as amide and amine groups will have previously formed hydrogen bonds with polar side chains on the outside of protein A/G [24].

In the case of the commercial Ag/Ac elution buffer pH 6.6 with high salt content, the high salt content, but not the pH value, may explain the results. The high salinity creates an environment of high ion concentration capable of interacting with any charge density group, disrupting the hydrophobic bonds between protein A/G and antibody and the hydrogen bonds between protein A/G and nylon, as well as the bare nylon nanofibres.

### Results of stability study

**Figure 7:**
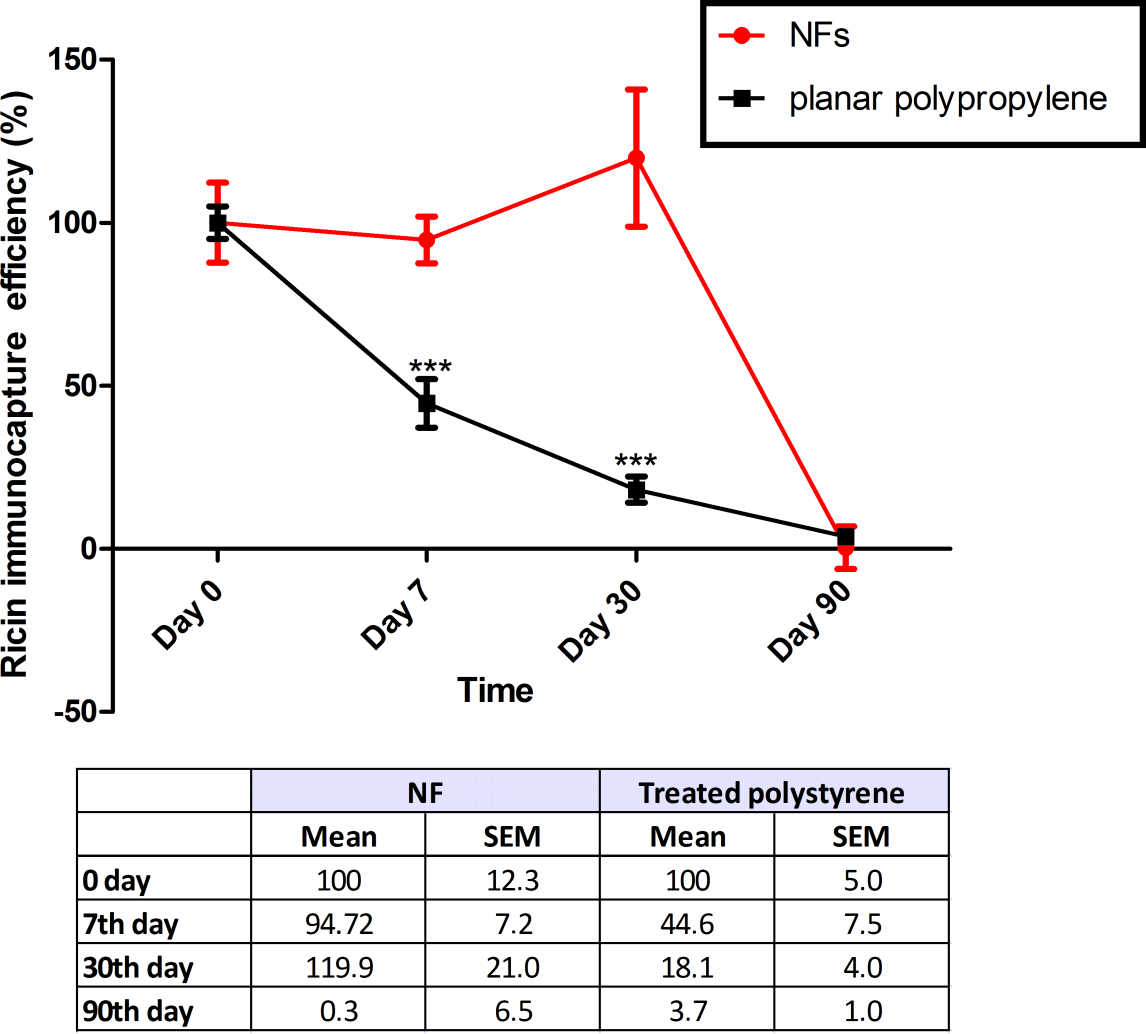
Fluorescence of ricin immunocaptured by the immunocapture system immobilised on both NFs and a specialised polypropylene ELISA microplate as function of the time, up to 90 days. Data are expressed as percentage of fluorescence of immunocaptured ricin on day 0, *n* = 4. Two-way ANOVA. Difference between the immunocapture system in NF and 96-well microplate, for each time: ***p < 0.001.

As this system is designed to be used for the on-site detection of biological agents, we wanted to conduct a stability study using a potential biological warfare agent, that is, ricin. The NFs allow the immunocapture capability of the system to remain absolutely intact for one month without the use of any preservative. In contrast, a polypropylene microplate specifically designed to optimise an enzyme-linked immunosorbent assay showed a decreasing immunocapture capability such that seven days after the immunocapture system was assembled, only 44.6% of ricin was immunocaptured compared to the initial measurement result (day 0); after 30 days, only 18.1% was detected (Figure 7). Two-way ANOVA showed these differences to be statistically significant.

### Discussion of stability study

As described by Feng et al. [27], hydrogen-bonded organic frameworks allow enzymes to diffuse into the pores, providing an additional layer of protection against denaturation factors. Since hydrogen bonds are formed between protein A/G and nylon, it is understandable that a three-dimensional nylon structure, such as the nanofibres, would provide more hydrogen bonds as attachment points than a planar surface, allowing the attached protein to be better protected.

## Conclusion

In summary, NFs with protein A/G are capable to be reused in a new immunocapture system, as long as the stripping treatment is carried out with glycine buffer pH 2.5. After treatment with this buffer, protein A/G is separated from antibody but not from the NFs, and no damage in its antibody binding capability was found.

This allows the system to be very cost-effective, not only because NFs can be used again, but also the previously anchored protein A/G. It reduces not only the cost, but also the time needed to provide a new immunocapture system ready to use. In addition, because the NFs protects the immunocapture system better than a planar surface specialised for anchoring antibodies, they allow the immunocapture system to extend its shelf life.

## Experimental

### Chemicals

**Figure 8:**
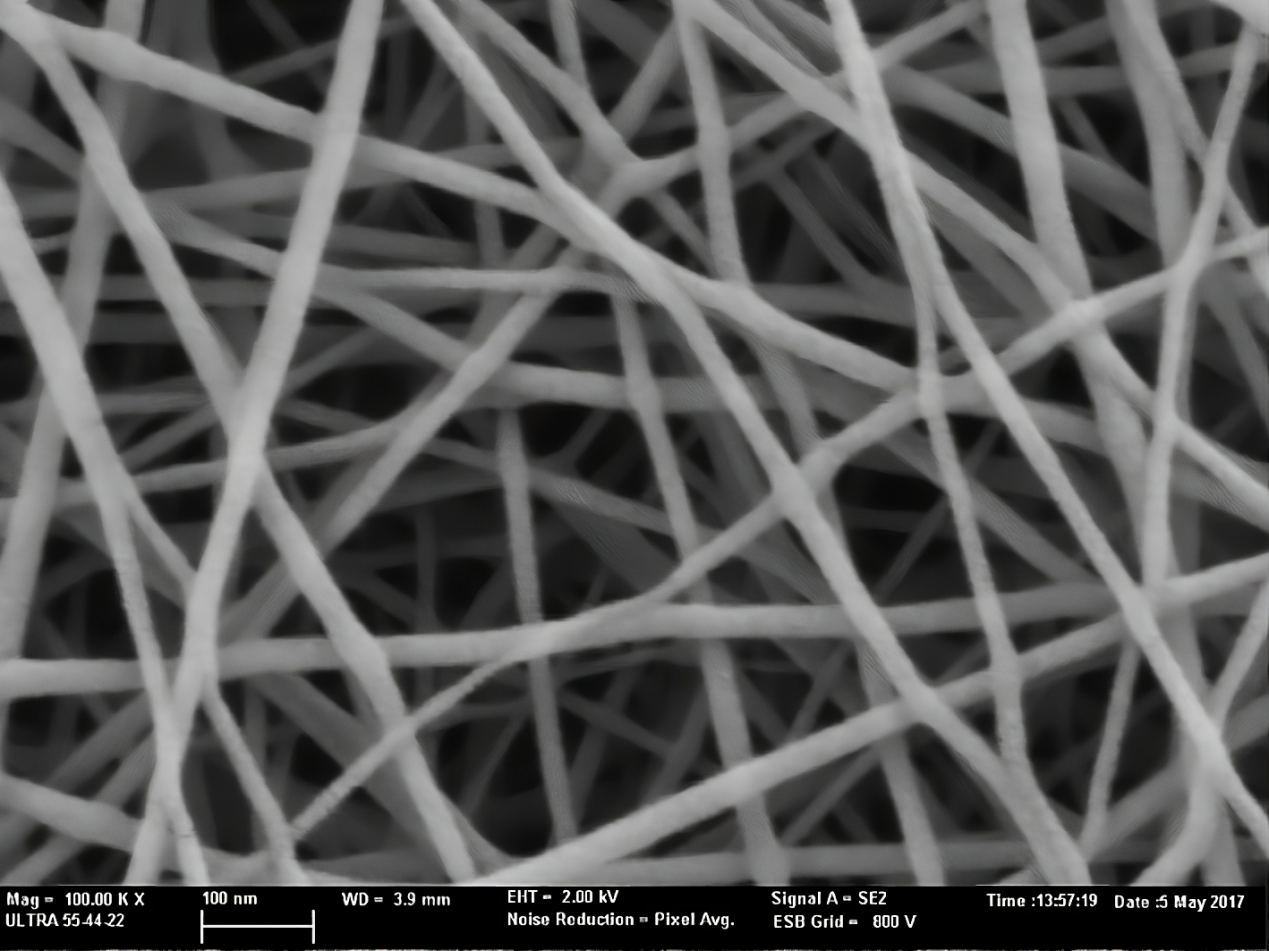
Field-emission scanning electron microscopy image of NFs.

PA6 was made by electrospinning by Tecnalia Research & Innovation, the composition of the ultrathin NFs was purchased from BASF (Ultramid^®^ B24 N 03). The NF manufacturing procedure was described in previous publications [17–19,28–29]. The average diameter of the NFs was 23 ± 5.8 nm, determined using the “ImageJ” analysis software (Figure 8).

NFs were cut in 4 × 4 mm^2^ samples to be placed and assayed in 96-well microplates. Nunc MaxiSorp^®^ flat-bottom microplates were used in the stability assay. Ricin was obtained from Robert Koch Institute. FITC-labelling kit and LYNX Rapid HRP antibody conjugation kit were purchased from BioRad, Spain. The anti-ricin antibody is an in-house-manufactured mouse antibody, made in collaboration with the National Center for Biotechnology (CNB) – CSIC. 10-Acetyl-3,7-dihydroxyphenoxazine (ADHP, Ampliflu) was used as a fluorogenic substrate for horseradish peroxidase (HRP) (Sigma-Aldrich). BSA, from Sigma-Aldrich, labelled with RPE was selected as toxin surrogate. FITC-labelled sheep polyclonal antibody against BSA was purchased from Thermo Fisher Scientific Inc. The blocking buffer was phosphate-buffered saline (PBS) with casein (Pierce). Solvents and additives were purchased from Aldrich (Spain). PBS was purchased from Fisher Scientific.

### Nanofiber reusability study

#### Immunocapture protocol

The immunocapture protocol used was published in 2018, as mentioned in the Introduction section [18]. It consisted of a well-oriented antibody immobilisation system containing the intermediate protein A/G. Briefly, NFs were placed in the microwells of a 96-well microplate, previously blocked with PBS–casein. In order to achieve a well-oriented antibody immobilisation, protein A/G (10 μL 100 μg/mL in PBS) was added to each NF sample and incubated overnight at 4 °C, followed by a blocking step with PBS–casein. Then, a FITC-labelled antibody against BSA was immobilized on the surface of the NFs containing protein A/G through 1 h of incubation. Then, RPE-labelled BSA (10 µL 100 µg/mL in blocking buffer) was immunocaptured by the anchored antibodies over a period of 1 h. Washing steps were carried out between each step in order to remove non-linked excess reagents. The fluorescence signals were measured using a Gemini XPS Microplate Reader (Molecular Devices) in RFU.

Anchored antibody was measured as FITC-fluorescence (λ_emission_ = 490 nm and λ_excitation_ = 521 nm) after incubation and subsequent wash, divided by the FITC fluorescence obtained just before antibody incubation (autofluorescence of the system).

Immunocaptured BSA was measured as RPE fluorescence (λ_emission_ = 495 nm and λ_excitation_ = 521 nm) after incubation and subsequent wash, divided by the RPE autofluorescence obtained just before antibody incubation.

Since a lot of handling is required, BSA was used as a toxin surrogate in “Nanofibre reusability study” because of safety and economic considerations.

#### Stripping treatments

Since we wanted to evaluate the role played by the pH value, three different pH buffers from acidic to basic were assayed. We used a Thermo Scientific™ Pierce™ Gentle Ag/Ab elution buffer pH 6.6, a glycine buffer pH 2.5 containing 200 mM glycine in PBS, and an ammonium hydroxide buffer pH 11 containing 1 N NH_4_OH in PBS (the latter two chemicals were purchased from Sigma-Aldrich).

The stripping protocol using any buffer was as follows: Stripping buffer was added (200 µL per NF sample) and incubated at room temperature for 10 min two times. The stripping buffer was removed from the nanofibers, and the NFs were washed with PBS (adding and incubating for 10 min two times). The two stripping buffer steps were repeated and three 5 min PBS wash steps took place after them (adapted from *abcam* stripping protocols [30]).

#### Reconstructing of the immunocapture system

The immunocapture systems were rebuilt as described above. In order to study how each treatment affects both immunocapture system and nanofibers, several groups were assayed: **Group 1:** complete immunocapture system (protein A/G + antibody-FITC + BSA-RPE) without stripping treatment (group 1). **Group 2:** complete immunocapture system (protein A/G + antibody-FITC + BSA-RPE) with stripping treatment. **Group 3:** complete immunocapture system (protein A/G + antibody-FITC + BSA-RPE), then stripping treatment and complete rebuild of the immunocapture system (protein A/G + antibody-FITC + BSA-RPE) afterward. **Group 4:** immunocapture system without BSA-RPE, then stripping treatment and only BSA-RPE added afterward. **Group 5:** only protein A/G anchored to NFs, then stripping treatment and only antibody-FITC incubation and BSA-RPE added afterward. **Group 6:** only bare NFs undergoing stripping treatment and complete rebuild of the immunocapture system (protein A/G + antibody-FITC + BSA-RPE) afterward.

Fluorescence of both anchored FITC-antibody and immunocaptured BSA-RPE was measured as described above. Results are shown as percentage fluorescence of the complete immunocapture system compared to the group 1, which is the 100% value. Data were statistically analysed by two two-way analysis of variance (ANOVA) using GraphPad Prims 5 Software.

### Stability study

Since less handling is required in this study, ricin is used as toxin instead of a surrogate as BSA.

#### Immunocapture system in stability study

The immunocapture system was similar as one described above. Briefly, NFs were placed in the microwells of a 96-well microplate, previously blocked with PBS–casein. Protein A/G (10 μL 100 μg/mL in PBS) was added to each NF sample surface and incubated over night at 4 °C, followed by a blocking step with PBS–casein. The control planar surface group was incubated with protein A/G overnight and then blocked with PBS–casein. Since only the immunocapture capability was measured in this study, nonlabelled in-house antibody (10 µL 500 µg/mL) against ricin was incubated for 1 h at room temperature. Then, biotin-labelled ricin (1 µL 1 mg/mL in blocking buffer) was immunocaptured by the anchored antibodies over 1 h of incubation. Biotin-ricin was added to both NF and microplate immunocapture systems at different times: day 0 (immediately after antibody anchoring; it is considered the reference value) and 7 days, 30 days, and 90 days after antibody anchoring, bringing the immunocapture systems to room temperature only covered by aluminum foil. Washing steps were carried out between each step above in order to remove non-linked excess reagents. Peroxidase-labelled streptavidin was added to detect immunocaptured ricin through biotin and streptavidin binding. A fluorescent peroxidase substrate (ADH, Ampliflu) was added, and the fluorescence was measured (λ_emission_ = 530 nm and λ_excitation_ = 590 nm). This value was divided by the fluorescence obtained just before incubation with biotin-labelled ricin (system autofluorescence). The results are expressed as a percentage of the fluorescence of immunocaptured ricin in the initial immunocapture system (day 0).

## Considerations

(1) The samples must be dissolved in buffer with a physiological pH value before testing them. (2) The sensing method used was fluorescence as it is a simple method that does not require any additional steps for its determination. However, even though the aim of the study was to evaluate the reusability and stability of NFs, the sensitivity of the system could be improved by using another more accurate sensing system. (3) Because of the pore size of the nanofibres, they cannot be used for the detection of bacteria, rickettsiae, or fungi (i.e., they cannot be used for the detection of prokaryotic or eukaryotic cells). They could, therefore, be used for the determination of exogenously produced biotoxins and virulence factors, as well as for the detection of viruses and biomarkers in clinical samples (e.g., hormones and biomolecules). (4) The data could be generalised not only for the measurement of warfare agents, but also for the diagnosis of water and food contamination and for the clinical diagnosis of infectious agents and biomarkers.
